# External validation of the modified sepsis renal angina index for prediction of severe acute kidney injury in children with septic shock

**DOI:** 10.1186/s13054-023-04746-6

**Published:** 2023-11-28

**Authors:** Natalja L. Stanski, Rajit K. Basu, Natalie Z. Cvijanovich, Julie C. Fitzgerald, Michael T. Bigham, Parag N. Jain, Adam J. Schwarz, Riad Lutfi, Neal J. Thomas, Torrey Baines, Bereketeab Haileselassie, Scott L. Weiss, Mihir R. Atreya, Andrew J. Lautz, Basilia Zingarelli, Stephen W. Standage, Jennifer Kaplan, Lakhmir S. Chawla, Stuart L. Goldstein

**Affiliations:** 1 Division of Critical Care Medicine, Cincinnati Children’s Hospital Medical Center, 3333 Burnet Ave MLC 2005, Cincinnati, OH 45229 USA; 2 Department of Pediatrics, University of Cincinnati College of Medicine, 3230 Eden Ave, Cincinnati, OH 45267 USA; 3 Division of Critical Care Medicine, Ann & Robert Lurie Children’s Hospital of Chicago, Northwestern University, 225 E. Chicago Ave, Chicago, IL 60611 USA; 4 UCSF Benioff Children’s Hospital Oakland, 747 52nd Ave, Oakland, CA 94609 USA; 5 Children’s Hospital of Philadelphia, 3401 Civic Center Blvd, Philadelphia, PA 19104 USA; 6 Akron Children’s Hospital, 214 W Bowery St., Akron, OH 44308 USA; 7 Texas Children’s Hospital and Baylor College of Medicine, 6621 Fannin Street, Houston, TX 77030 USA; 8 Children’s Hospital of Orange County, 1201 W La Veta Ave, Orange, CA 92868 USA; 9 Riley Hospital for Children, 705 Riley Hospital Drive, Indianapolis, IN 46202 USA; 10 Penn State Health Children’s Hospital, 600 University Drive, Hershey, PA 17033 USA; 11 University of Florida Health Shands Children’s Hospital, 1600 South West Archer Rd, Gainesville, FL 32608 USA; 12 Lucile Packard Children’s Hospital Stanford, 725 Welch Rd, Palo Alto, CA 94304 USA; 13 Nemours Children’s Health, 1600 Rockland Rd, Wilmington, DE 19803 USA; 14 Department of Medicine, Veterans Affairs Medical Center San Diego, 3350 La Jolla Village Drive, San Diego, CA 92161 USA; 15 Division of Nephrology and Hypertension, Cincinnati Children’s Hospital Medical Center, 3333 Burnet Ave, Cincinnati, OH 45229 USA

**Keywords:** Acute kidney injury, Sepsis, Shock, Precision medicine, Prognostic enrichment, Prediction, Pediatrics

## Abstract

**Background:**

Acute kidney injury (AKI) occurs commonly in pediatric septic shock and increases morbidity and mortality. Early identification of high-risk patients can facilitate targeted intervention to improve outcomes. We previously modified the renal angina index (RAI), a validated AKI prediction tool, to improve specificity in this population (sRAI). Here, we prospectively assess sRAI performance in a separate cohort.

**Methods:**

A secondary analysis of a prospective, multicenter, observational study of children with septic shock admitted to the pediatric intensive care unit from 1/2019 to 12/2022. The primary outcome was severe AKI (≥ KDIGO Stage 2) on Day 3 (D3 severe AKI), and we compared predictive performance of the sRAI (calculated on Day 1) to the original RAI and serum creatinine elevation above baseline (D1 SCr > Baseline +). Original renal angina fulfillment (RAI +) was defined as RAI ≥ 8; sepsis renal angina fulfillment (sRAI +) was defined as RAI ≥ 20 *or* RAI 8 to < 20 with platelets < 150 × 10^3^/µL.

**Results:**

Among 363 patients, 79 (22%) developed D3 severe AKI. One hundred forty (39%) were sRAI + , 195 (54%) RAI + , and 253 (70%) D1 SCr > Baseline + . Compared to sRAI-, sRAI + had higher risk of D3 severe AKI (RR 8.9, 95%CI 5–16, *p* < 0.001), kidney replacement therapy (KRT) (RR 18, 95%CI 6.6–49, *p* < 0.001), and mortality (RR 2.5, 95%CI 1.2–5.5, *p* = 0.013). sRAI predicted D3 severe AKI with an AUROC of 0.86 (95%CI 0.82–0.90), with greater specificity (74%) than D1 SCr > Baseline (36%) and RAI + (58%). On multivariable regression, sRAI + retained associations with D3 severe AKI (aOR 4.5, 95%CI 2.0–10.2, *p* < 0.001) and need for KRT (aOR 5.6, 95%CI 1.5–21.5, *p* = 0.01).

**Conclusions:**

Prediction of severe AKI in pediatric septic shock is important to improve outcomes, allocate resources, and inform enrollment in clinical trials examining potential disease-modifying therapies. The sRAI affords more accurate and specific prediction than context-free SCr elevation or the original RAI in this population.

**Supplementary Information:**

The online version contains supplementary material available at 10.1186/s13054-023-04746-6.

## Background

Septic shock accounts for 10% of pediatric intensive care unit (PICU) admissions worldwide and is associated with high rates of morbidity and mortality [1–3]. One in five children with septic shock develops severe acute kidney injury (AKI) (defined as Kidney Disease Improving Global Outcomes [KDIGO] Stage 2 or 3) which confers even higher risk for poor outcomes, including greater than five times the odds of mortality and risk for new disability in survivors [4–6]. Only supportive therapies are available to treat severe AKI once present, including kidney protection strategies (e.g., avoidance of excessive fluid accumulation, maintenance of adequate kidney perfusion, and judicious use of nephrotoxic medications) and provision of kidney replacement therapy (KRT) when necessary. These therapies may be more successful in mitigating AKI if implemented early, in at-risk children, and thus strategies for identification of children with septic shock at risk for severe AKI development and/or persistence are necessary to improve care and outcomes for these children.

**Fig. 1:**
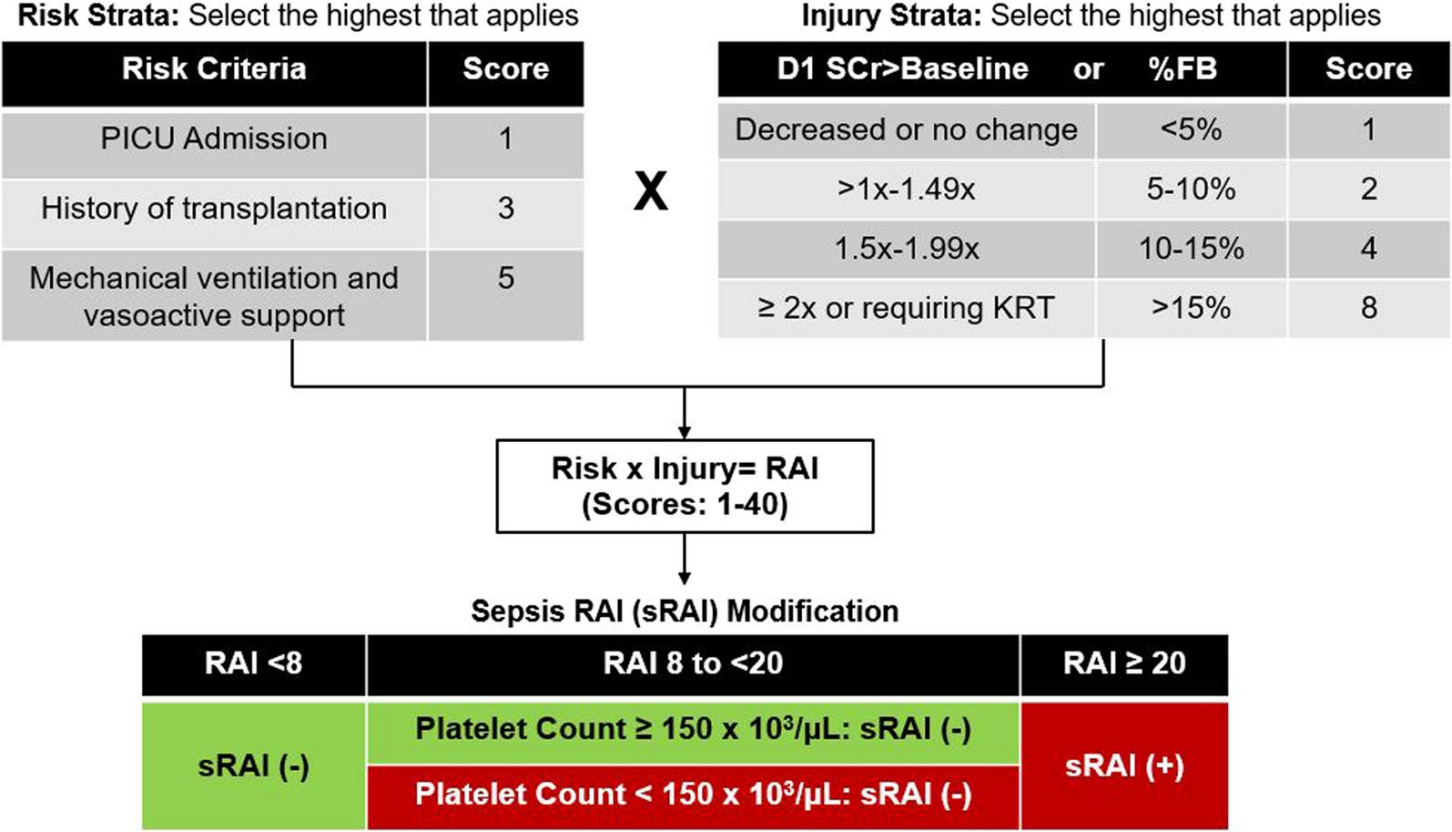
Modified sepsis renal angina index. First, the RAI score is calculated as the product of the highest risk and injury strata, resulting in a score between 1 and 40. Next, the sepsis modification is applied as follows: (1) if the patient has an RAI score < 8, they are deemed low risk and not predicted to have D3 severe AKI (sRAI-, green); (2) if the patient has an RAI score ≥ 20, they are deemed high risk and predicted to have D3 severe AKI (sRAI + , red); (3) if the patient has an intermediate RAI score (8 to < 20), platelet count is considered for further risk stratification. Patients with platelet count ≥ 150 × 10^3^/µL are deemed low risk and not predicted to have D3 severe AKI (sRAI-, green), while those with platelet count < 150 × 10^3^/µL are deemed high risk and predicted to have D3 severe AKI (sRAI + , red). SCr > Baseline: degree of serum creatinine elevation above baseline on Day 1 of septic shock; %FB: cumulative percent fluid balance on Day 1 of septic shock; sRAI: modified sepsis renal angina index; RAI: Renal angina index; D3 severe AKI: severe acute kidney injury on Day 3 of septic shock

The renal angina index (RAI) is a validated scoring tool that combines readily available demographic and clinical information from the first 12 h of PICU admission to predict risk for severe AKI 72 h later (Fig. 1) [7, 8]. The RAI has been studied across a variety of settings in general populations of critically ill children with consistently good predictive performance, including an area under the receiver operating curve [AUROC] of 0.88 (95%CI 0.85–0.910), 85% sensitivity, and 79% specificity in a recent meta-analysis of 11 studies comprising 3700 patients [7, 9–12]. However, children with septic shock often have 1) elevated serum creatinine [SCr] in the setting of hypovolemia, 2) received large-volume fluid resuscitation, and 3) require mechanical ventilation and vasoactive medications 12 h after PICU admission, all of which contribute to a positive RAI score when present (RAI ≥ 8, Fig. 1). These unique clinical features led us to hypothesize that the predictive performance of the RAI— and in particular its specificity— would be diminished in children with septic shock, which was validated by our previous pilot work demonstrating only 54% specificity in this population [13]. To address this issue, we recalibrated the RAI for children with septic shock (modified sepsis RAI [sRAI]) (Fig. 1) [13]. Though the sRAI performed well in the derivation cohort (AUROC 0.90, 95%CI 0.86–0.93; 95% sensitivity, 69% specificity, 39% positive predictive value [PPV], 99% negative predictive value [NPV]) [13], it has not yet been tested in a separate cohort of children with septic shock.

Herein, we assess sRAI performance for prediction of severe AKI at Day 3 (D3 severe AKI) in a new cohort of children from a multicenter study of pediatric septic shock. We hypothesized that: 1) the sRAI predictive performance would be superior to both early (Day 1) context-free SCr elevation above baseline *and* the original RAI; and 2) fulfillment of sepsis renal angina criteria (sRAI +) would be independently associated with D3 severe AKI and need for KRT in the first week of septic shock.

## Methods

### Study design and patient selection

We performed a secondary analysis of a multicenter, prospective, observational cohort study of children with septic shock aged one week to 18 years admitted to 10 PICUs across the USA from January 2019 to December 2022. The original study protocol has been previously described in detail [14] and was approved by the Institutional Review Boards at each participating site prior to patient enrollment. Patients meeting criteria for pediatric septic shock (Additional file 1) were enrolled in the original study after obtaining informed consent from parents or legal guardians; the only exclusion criterion was the inability to obtain informed consent. After enrollment, clinical and laboratory data were collected daily during PICU admission for up to seven days, with outcomes tracked for 28 days. Patients from the original study (*n* = 384) were excluded from our analysis if they had end stage kidney disease *or* pre-existing kidney disease without a known baseline SCr (*n *= 14) or if they were missing SCr data from Day 1 or Day 3 (*n* = 7).

### *RAI calculation and determination of sepsis renal angina fulfillment (sRAI* +*)*

The RAI was calculated for each patient using clinical and demographic data from the first 24 h after meeting enrollment criteria for septic shock, with a score of ≥ 8 defining fulfillment of renal angina criteria (RAI +), as previously published (Fig. 1) [7, 8, 13]. Based on our RAI recalibration for septic shock [13], the presence or absence of sepsis renal anginal fulfillment (sRAI + or sRAI-, respectively) was subsequently determined using the original calculated RAI and Day 1 platelet count: (1) sRAI + : RAI ≥ 20 *or* RAI 8 to < 20 with platelet count < 150 × 10^3^/µL, and (2) sRAI-: RAI < 8 *or* RAI 8 to < 20 with platelet count ≥ 150 × 10^3^/µL (Fig. 1). Patients assigned sRAI + were predicted to have D3 severe AKI, while those assigned sRAI- were predicted not to have D3 severe AKI.

### Outcomes and Definitions

The primary outcome of interest was performance of sRAI to predict D3 severe AKI, defined as Kidney Disease Improving Global Outcomes (KDIGO) stage 2 AKI or higher using SCr criteria (SCr ≥ 2 × baseline) [15]. Baseline SCr was the lowest measured within three months prior to PICU admission; if no value was available, baseline SCr was imputed using an estimated glomerular filtration rate (eGFR) of 120 ml/min per 1.73 m^2^, as previously validated [16, 17]. In patients without either documented baseline SCr or height for body surface area calculation (*n* = 11), the age-based Pottel method was used to assign a baseline SCr [18]. Secondary outcomes of interest included need for KRT in the first week of septic shock, PICU free days (calculated as 28 – PICU length of stay; patients who died in PICU within the first 28 days were assigned “0”), and 28-day PICU mortality.

Day 1 fluid balance (%FB) was calculated using the following formula: %FB = [(Total Fluid In (L) – Total Fluid Out (L))/Admission Weight (kg)] × 100% [19, 20]. The degree of SCr elevation above baseline on Day 1 of septic shock (D1 SCr > Baseline) was calculated as a ratio of Day 1 measured SCr/Baseline SCr, as has been reported previously in pediatric RAI literature [7, 13]. As part of the original study, each patient had Day 1 serum collected for measurement of the PERSEVERE biomarkers, which were used to assign a validated, pediatric-sepsis specific mortality probability known as PERSEVERE-II [14, 21]. Severity of illness was also quantified at admission for septic shock using the validated Pediatric Risk of Mortality III score [22] and the day 1 vasoactive-inotropic score (D1 VIS) [23].

### Statistical analyses

Data were described using medians, interquartile ranges, frequencies, and percentages. Comparisons between groups were performed using relative risks, Wilcoxon rank sum, Chi-square, or Fisher’s exact test, as appropriate. ROC curves were generated to assess the predictive performance of the RAI, sRAI and D1 SCr > Baseline for D3 severe AKI, with AUROCs compared using the DeLong test. Sensitivities, specificities, PPVs, NPVs, positive likelihood ratios (+ LRs) and negative likelihood ratios (-LRs) were generated for sRAI + , D1 SCr > Baseline + (i.e., having D1 SCr > Baseline > 1), and RAI + (i.e., at the typical threshold of ≥ 8 without the platelet modifier). Multivariable logistic regression was performed to assess the independent association between sRAI + and D1 SCr > Baseline + with the development of D3 severe AKI and need for KRT, after adjustment for significant covariates identified on bivariate analysis comparing patients with and without D3 severe AKI (*p* < 0.15). Of note, covariates encompassed within the sRAI score (history of transplant, receipt of vasoactive medications, receipt of mechanical ventilation, %FB and platelet count) were a priori excluded from the regression analyses due to the possibility of collinearity. Additionally, patients requiring KRT on Day 1 (*n* = 26) were excluded from the regression analyses due to the potential impact on D1 SCr > Baseline values. A *p*-value < 0.05 was considered statistically significant. All statistical analyses were performed using Sigmaplot 15.0 (Systat Software Inc., Palo Alto, CA) and R Version 4.2.2 (R Foundation for Statistical Computing, Vienna, Austria).

## Results

### Patient characteristics

The original study cohort included 384 children admitted to PICU with septic shock. After exclusion of patients with end stage kidney disease *or* pre-existing kidney disease without a known baseline SCr (*n* = 14) and those missing SCr data from Day 1 or Day 3 (*n* = 7), 363 patients were included in the current analysis (Additional file 2: Figure S1), all of whom had complete data on Day 1, Day 3, and Day 28. One hundred forty patients (39%) were sRAI + on Day 1 of septic shock. Table 1 outlines clinical, demographic and outcome data for the cohort by presence or absence of sepsis renal angina fulfillment. sRAI + patients more commonly had: (1) a history of transplant (solid organ or hematopoietic stem cell), (2) higher severity of illness by both PRISM III and PERSEVERE-II, and (3) higher incidence of requiring vasoactive support and invasive mechanical ventilation on Day 1, as well as higher intensity of vasoactive support by VIS. The sRAI + cohort had increased risk for D3 severe AKI (RR 8.9, 95%CI 5.0–16.0, *p* < 0.001), KRT use in the first week of septic shock (RR 18, 95%CI 6.6–49, *p* < 0.001), and 28-day mortality (RR 2.5, 95%CI 1.2–5.5, *p* = 0.013), as well as fewer PICU free days (14.5 days [0–23] *vs*. 25 days [19–26], *p* < 0.001). (Additional file 3: Tables S1 and S2) outline the incidence of D3 severe AKI and KRT use by each possible RAI score, sRAI designation (sRAI + /sRAI-) and by individual score components.

**Table 1:** Demographic, clinical and outcome characteristics of the cohort by presence or absence of sepsis renal angina (sRAI) fulfillment on Day 1

	All	sRAI-	sRAI +	*p*
N (%cohort)	363	223 (61)	140 (39)	–
Age, years	9.6 (3.3–16.3)	10.3 (4.1–16.3)	8.6 (2.2–15.9)	0.06
Male sex, n (%)	187 (52)	106 (48)	81 (58)	0.06
History of Transplant	34 (9)	10 (4)	24 (17)	< 0.001
*Severity of Illness*				
PRISM III	8 (5–13)	7 (3–10)	12 (8–17)	< 0.001
PERSEVERE-II Mortality Probability	0.007 (0.007–0.189)	0.007 (0.007–0.019)	0.167 (0.007–0.325)	< 0.001
Baseline SCr, mg/dl	0.36 (0.24–0.49)	0.38 (0.26–0.52)	0.33 (0.22–0.47)	0.012
Baseline Known, n (%)	205 (56)	127 (57)	78 (56)	0.82
D1 Vasoactive Medications, n (%) D1 VIS	268 (74) 7.0 (0–18)	138 (62) 4.0 (0–10)	130 (93) 14.5 (6–27.8)	< 0.001 < 0.001
D1 Mechanical Ventilation, n (%)	202 (56)	87 (39)	115 (82)	< 0.001
D1 Fluid Balance (%FB)	3.9 (1.3–7.1)	3.4 (1.1–6.3)	4.9 (1.7–8.9)	0.001
D1 SCr > Baseline	1.3 (0.96–2.1)	1.1 (0.9–1.4)	2.2 (1.3–3.6)	< 0.001
D1 Platelet Count (× 10^3^/uL)	153 (70–265)	188 (105–305)	98 (45–170)	< 0.001
D3 Severe AKI, n (%)	79 (22)	12 (5)	67 (48)	< 0.001
Relative Risk Severe AKI	–	–	RR 8.9 (5–16)	< 0.001
D1-7 KRT Use, n (%)	49 (13)	4 (2)	45 (32)	< 0.001
Relative Risk KRT Use	–	–	RR 18 (6.6–49)	< 0.001
PICU Free Days	22 (10–26)	25 (19–26)	14.5 (0–23)	< 0.001
28-day Mortality, n (%)	26 (7.2)	10 (4)	16 (11)	0.013
Relative Risk Mortality	–	–	RR 2.5 (1.2–5.5)	0.013

sRAI—sepsis renal angina index; PRISM III—Pediatric Risk of Mortality III; SCr—serum creatinine; D—day; SCr > Baseline—degree of serum creatinine elevation above baseline (D1 SCr divided by Baseline SCr); AKI—acute kidney injury; KRT—kidney replacement therapy; RR—relative risk; VIS—vasoactive-inotropic score

Data for categorical variables are reported as n (%) and continuous variables as median (IQR)

Seventy-nine patients (22%) developed D3 severe AKI. Demographic, clinical and outcome variables for patients with and without D3 severe AKI are shown in Table 2. On Day 1 of septic shock, patients who developed D3 severe AKI had: (1) higher severity of illness by PRISM III and PERSEVERE-II, (2) higher rates of vasoactive medication use and invasive mechanical ventilation, (3) higher intensity of vasoactive support by VIS and 4) higher %FB, greater degree of elevation of SCr > Baseline, and lower median platelet count (Table 2). Patients with D3 severe AKI also had fewer PICU free days (4 days [0–20] *vs*. 24 days [16–26], *p* < 0.001) and great risk for mortality (RR 4.9, 95%CI 2.4–10.2, *p* < 0.001).

**Table 2:** Demographic, clinical and outcome characteristics based on the presence or absence of severe acute kidney injury on day 3

	No D3 Severe AKI	D3 Severe AKI	*p*-value
N (%cohort)	284 (78)	79 (22)	–
Age, years	9.6 (3.8–16.3)	10.3 (2.1–16.5)	0.54
Male sex, n (%)	144 (51)	43 (54)	0.56
History of Transplant	16 (6)	18 (23)	< 0.001
*Severity of Illness*			
PRISM III	8 (4–12)	13 (8–17)	< 0.001
PERSEVERE-II Mortality Probability	0.007 (0.007–0.167)	0.189 (0.007–0.333)	< 0.001
Baseline SCr, mg/dl	0.37 (0.26–0.5)	0.30 (0.2–0.46)	0.010
Baseline known, n (%)	149 (52)	56 (71)	0.003
D1 Vasoactive Medications, n (%)	198 (70)	70 (89)	< 0.001
D1 VIS	5.0 (0–14)	17.0 (5.5–36.5)	< 0.001
D1 Mechanical Ventilation, n (%)	142 (50)	60 (76)	< 0.001
D1 Fluid Balance (%FB)	3.5 (1.1–6.5)	4.9 (2.2–9.1)	0.005
D1 SCr > Baseline	1.2 (0.9–1.6)	2.8 (1.7–4.4)	< 0.001
D1 Platelet Count (× 10^3^/uL)	172 (88–273)	91 (39–227)	< 0.001
PICU Free Days	24 (16–26)	4 (0–20)	< 0.001
28-day Mortality, n (%)	11 (4)	15 (19) RR 4.9 (2.4–10.2)	< 0.001 < 0.001

PRISM III—Pediatric Risk of Mortality III; SCr—serum creatinine; D—day; SCr > Baseline—degree of serum creatinine elevation above baseline (D1 SCr divided by Baseline SCr); AKI—acute kidney injury; RR—relative risk; VIS—vasoactive-inotropic score

Data for categorical variables are reported as n (%) and continuous variables

### Assessment of sRAI for prediction of D3 severe AKI and comparison to context-free serum creatinine elevation and original RAI

Table 3 outlines clinical, demographic and outcome variables for sRAI + patients compared to those who were D1 SCr > Baseline + and RAI + , as well as the predictive performance of each for D3 severe AKI. The sRAI performed well to predict D3 severe AKI, with AUROC 0.86 (95%CI 0.82–0.90), sensitivity 85% (95%CI 75–92), specificity 74% (95%CI 69–79), PPV 48% (95%CI 39–56), and NPV 95% (95%CI 91–97).

**Table 3:** Clinical, demographic and outcome variables and predictive performance of day 1 sepsis renal angina fulfillment (sRAI +) compared to degree of serum creatinine elevation above baseline (D1 SCr > Baseline) and original renal angina fulfillment (RAI +)

	sRAI + (*n* = 140)	D1SCr > Baseline + (*n* = 253)	*p*-value sRAI + vs. D1SCr +	RAI + (*n* = 195)	*p-*value sRAI + vs. RAI +
D3 Severe AKI, n (%)	67 (48)	71 (28)	< 0.001	76 (39)	0.11
RR vs. D1 SCr > Baseline +	RR 1.7 (1.3–2.2)	–	< 0.001	–	–
RR vs. RAI +	RR 1.2 (0.96–1.6)	–	–	–	0.11
*D3 Severe AKI Prediction*					
AUROC	0.86 (0.82–0.90)	0.82 (0.76–0.88)	0.19	0.86 (0.82–0.90)	–
Sensitivity, %	85 (75–92)	90 (81–95)		96 (89–99)	
Specificity, %	74 (69–79)	36 (30–42)		58 (52–64)	
PPV, %	48 (39–56)	28 (23–34)		39 (32–46)	
NPV, %	95 (91–97)	93 (86–97)		98 (94–99)	
+ LR	3.3 (2.7–4.1)	1.4 (1.3–1.6)		2.3 (2.0–2.7)	
-LR	0.2 (0.1–0.4)	0.28 (0.1–0.6)		0.07 (0.02–0.2)	
D1-7 KRT Use. n (%)	45 (32)	42 (12)	< 0.001	46 (24)	0.08
RR vs. D1 SCr > Baseline +	RR 1.9 (1.3–2.8)	–	< 0.001	–	–
RR vs. RAI +	RR 1.4 (0.96–1.9)	–	–	–	0.08
PICU Free Days	14.5 (0–23)	23 (11–26)	< 0.001	18 (0,24)	0.15
28-day Mortality, n (%)	16 (11)	18 (7)	0.15	20 (10)	0.73
RR vs. D1 SCr > Baseline +	RR 1.6 (0.8–3)	–	0.15	–	–
RR vs. RAI +	RR 1.1 (0.6–2.1)	–	–	–	0.73

sRAI +—sepsis renal angina fulfillment; D1SCr > Baseline +—serum creatinine above baseline on Day 1; RAI +—renal angina fulfillment; PRISM III—Pediatric Risk of Mortality III; SCr—serum creatinine; D—day; AKI—acute kidney injury; KRT—kidney replacement therapy; RR—relative risk

Data for categorical variables are reported as n (%) and continuous variables as median (IQR)

While the predictive performance of the sRAI compared to D1 SCr > Baseline by AUROC (0.86 [0.82–0.90] *vs*. 0.82 [0.76–0.88], *p* = 0.19) did not differ, sRAI had higher specificity (74% *vs.* 36%), PPV (48% *vs*. 28%) and + LR (3.3 *vs*. 1.4) (Table 3). Compared to D1 SCr > Baseline + patients, sRAI + patients had higher risk of D3 severe AKI (RR 1.7, 95%CI 1.3–2.2, *p* < 0.001), higher risk of requiring KRT in the first week of septic shock (RR 1.9, 95%CI 1.3–2.8, *p* < 0.001), and fewer PICU free days (14.5 days [0–23] *vs*. 23 days [11–26], *p* < 0.001).

One hundred ninety-five patients (54%) met the original criteria for renal angina fulfillment (RAI +), compared to 140 (39%) who met sRAI + criteria. D3 severe AKI occurred in 76 of 195 RAI + patients (39%) compared to 67 of 140 sRAI + patients (48%) (*p* = 0.11). While sRAI + was less sensitive for D3 severe AKI prediction (85% *vs*. 96%), sRAI + was more specific (74% *vs.* 58%), with higher PPV (48% *vs.* 39%) and relatively preserved NPV (95% *vs* 98%). There were no differences in PICU free days, risk of KRT or mortality, between sRAI + and RAI + patients (Table 3). Additional file 2: Figure S2 further illustrates the incidence of D3 severe AKI using each predictive tool and highlights the additive benefit of the sRAI modification above the original RAI with regard to PPV.

### Examining association of sRAI + with outcomes

On multivariable logistic regression, sRAI + retained associations with D3 severe AKI (adjusted OR 4.54, 95%CI 2.03–10.2, *p* < 0.001) and need for KRT in the first week of septic shock (adjusted OR 5.6, 95%CI 1.48–21.5, *p* = 0.01), after adjustment for other significant covariates identified on bivariate analysis (PRISM III, D1 VIS, PERSEVERE-II, baseline SCr) (Table 4). While D1 SCr > Baseline + also retained significant associations with D3 severe AKI, similar findings were not seen with need for KRT in the first week (Table 4).

**Table 4:** Multivariable logistic regression examining the association between sepsis renal angina fulfillment (sRAI +), serum creatinine above baseline on day 1 (D1 SCr > Baseline +), severity of illness and baseline creatinine used on the presence of severe acute kidney injury on day 3 and need for kidney replacement therapy

Outcome	Variable	Adjusted OR	95% CI	*P*
D3 Severe AKI	PRISM III PERSEVERE-II* Baseline SCr D1 VIS sRAI + D1 SCr > Baseline +	1.00 1.33 0.09 1.02 4.54 3.2	0.94–1.05 1.06–1.68 0.008–0.91 1.002–1.04 2.03–10.2 1.06–9.8	0.87 0.02 0.04 0.03 < 0.001 0.039
Need for KRT	PRISM III PERSEVERE-II* Baseline SCr D1 VIS sRAI + D1 SCr > Baseline +	0.92 1.67 1.76 1.03 5.65 0.97	0.85–0.99 1.2–2.3 0.06–48.0 1.01–1.05 1.48–21.5 0.24–3.98	0.036 0.002 0.74 0.002 0.011 0.96

*PERSEVERE-II Mortality Probability transformed by a factor of 10 for regression analyses

**Only included 337 patients (excluded those on KRT on Day 1)

PRISM III–Pediatric Risk of Mortality III; sRAI +—sepsis renal angina criteria fulfilled; D1 SCr > Baseline +—serum creatinine above baseline on Day 1; AKI—acute kidney injury; KRT—kidney replacement therapy; VIS—vasoactive-inotropic score

## Discussion

We assessed the performance of the modified sepsis renal angina Index (sRAI) for prediction of D3 severe AKI in a multicenter cohort of critically ill children with septic shock. Similar to our previous work [13], the sRAI demonstrated better specificity than the original RAI (74% *vs*. 58%) with modestly reduced sensitivity (85% *vs*. 96%) and improved predictive performance over context-free SCr changes alone, with more than twice the specificity and 20% higher PPV compared to D1 SCr > Baseline + . sRAI + was the strongest independent predictor of D3 severe AKI after adjustment for severity of illness and other covariates of interest, and unlike D1 SCr > Baseline + , retained an association with KRT in the first week of septic shock. Taken together, these data demonstrate the sRAI may serve as an early, reliable, and specific risk stratification tool for new or persistent severe AKI on Day 3 of pediatric septic shock, whose value is further underscored by its ease of calculation across a variety of settings using readily available clinical and laboratory data.

The ability to accurately assess children with septic shock early in PICU admission for risk of severe AKI development or persistence is vital, both for implementation of focused kidney protection strategies, and consideration of transfer to a higher level of care, if necessary (i.e., to KRT capable centers). In more resource-rich settings, early risk stratification can also guide further testing with additional biomarkers (e.g., urine neutrophil gelatinase-associated lipocalin [NGAL]) that may further delineate individual risk profile or be used to subgroup patients into unique subphenotypes of AKI which could guide management [24–27]. For example, a patient who is sRAI + and has an elevated urine NGAL can be identified as highest risk for D3 severe AKI and managed with more conservative fluid resuscitation, earlier vasoactive medication support, careful monitoring of nephrotoxic medications and consideration of earlier KRT to mitigate fluid accumulation, if necessary; conversely, a patient who is sRAI + but without urine NGAL elevation (or sRAI-) may be identified as lower risk and undergo standard management. This process of stepwise risk stratification has been successfully implemented in general populations of critically ill children at our center, with demonstrated improvements in AKI-related outcomes in the post-implementation period [28]. Importantly, such an approach may also be used to enrich clinical trials seeking to identify disease-modifying therapies for sepsis-associated AKI, increasing the likelihood of demonstrating benefit from a particular intervention or treatment should it exist. In each of these instances, increasing the pre-test probability for D3 severe AKI is important when considering costly interventions such as transfer to a higher level of care, earlier initiation of KRT, or additional biomarker measurement. Our data suggest that while just 22 out of 100 children with septic shock will have D3 severe AKI, the incidence is modulated using each of these tools to enrich the population at risk: 28 out of 100 children who are D1 SCr > Baseline + will have D3 severe AKI, compared to 39 out of 100 who are RAI + , and 48 out of 100 who are sRAI + . Thus, sRAI + identifies a subset of patients with the highest likelihood of D3 severe AKI, making it an important tool to aid in delegation of resources.

Similar to the prospective assessment of the RAI by Basu and colleagues [7], our results provide further confirmation that early context-free SCr elevation is an indiscriminate risk assessment tool for future AKI development or persistence (i.e., D3 severe AKI). Additionally, this finding appears to be even more prominent in children with septic shock, as 253 patients (70% of the cohort) had SCr elevation above baseline on Day 1 in this study compared to only 44% in the previous study of all-comers to the PICU [7]. This difference is perhaps unsurprising given the pathophysiology of septic shock, which often includes intravascular depletion, hypotension with decreased kidney perfusion, and a dysregulated host immune response to infection, all of which can drive early SCr elevation [24, 29, 30]. However, despite the differences in incidence of early SCr elevation between our septic shock cohort and the heterogeneous cohort of critically ill children studied by Basu et al., the incidence of D3 severe AKI was similar in both studies (22% *vs*. 23%, respectively) [7]. This finding highlights the need for additional tools like the sRAI to refine the risk assessment for severe AKI development or persistence beyond what is provided by early SCr elevation in children with septic shock.

While sRAI + improved the specificity of D3 severe AKI prediction in this cohort, it is important to note that RAI + also outperformed D1 SCr > Baseline + , with greater sensitivity (96% *vs.* 90%), specificity (58% *vs.* 36%), PPV (39% *vs*. 28%) and NPV (98% *vs*. 93%). Though our data suggest the sRAI provides the greatest predictive accuracy in children with septic shock [13], these findings are important to note for two main reasons. First, resource-limited centers without access to routine assessment of platelet count can alternatively use the original RAI as an improvement upon SCr-based risk assessment alone, as has been previously done [9, 10]. Second, we anticipate that implementation of the sRAI may be more difficult than the RAI due to inherent challenges in both recognition of sepsis (by clinicians and electronic health records) and its timing (i.e., a patient can develop sepsis at time points other than PICU admission), which currently represents an important barrier to its use at the bedside.

This study has several strengths. The cohort was large and multicenter, comprised of patients from multiple centers across the USA, increasing generalizability of the results. Our ability to utilize PERSEVERE-II mortality probability as an additional and specific marker of pediatric septic shock illness severity may improve our ability to quantify illness severity, thus enhancing the validity of our regression analyses. Unlike previous work, a substantial portion of the cohort (56%) had documented baseline SCr, improving the validity of SCr-defined AKI rates.

Our work also has important limitations. This is a secondary analysis of an observational study not designed to study the sRAI, and thus there is the possibility of unintended bias. As in our previous study [13], the RAI was calculated using data from the first 24 h of septic shock, as opposed to the precise 12 h timepoint previously validated [7]. Accurate urine output data were not available, raising the possibility that AKI rates were underestimated [31]. Additionally, details regarding the amount of fluid resuscitation received prior to PICU admission and enrollment were not available, and thus D1%FB may be underestimated. Finally, this study was performed at large, tertiary or quaternary medical centers in the USA, reducing the generalizability of findings in other settings and populations around the world. This is of particular importance given that children in resource-limited settings often present later in the course of illness, which may limit the utility of predictive tools like the sRAI. This is an important area of future research given the global impact of pediatric septic shock and sepsis-associated AKI.

## Conclusions

In conclusion, the modified sepsis RAI (sRAI) demonstrates good prospective performance for prediction of new or persistent D3 severe AKI in children with septic shock and is independently associated with adverse kidney outcomes including need for KRT. The accuracy of prediction and strength of these associations are superior to early context-free SCr elevation, and its performance offers improved specificity over the original RAI, though at the cost of modest reduction in sensitivity. Future prospective studies are needed to assess its practical implementation at the bedside.

### Supplementary Information

**Additional file 1**: Supplemental Methods. Septic shock criteria for original study.

**Additional file 2**:** Supplemental Figures**.** Figure S1.** CONSORT flow diagram for patient inclusion;** Figure S2.** Flow diagram of Day 1 acute kidney injury risk assessment tools and incidence of Day 3 severe acute kidney injury

**Additional file 3**:** Supplemental Tables. Table S1.** Distribution of outcomes by Renal Angina Index (RAI) Score andmodified sepsis RAI (sRAI) designation;** Table S2.** Distribution of Outcomes by Renal Angina Index (RAI) and modified sepsis RAI (sRAI) components.

## Data Availability

The datasets used and/or analyzed during the current study are available from the corresponding author on reasonable request.

## Abbreviations

PICU: Pediatric intensive care unit
AKI: Acute kidney injury
KRT: Kidney replacement therapy
RAI: Renal angina Index
sRAI: Modified sepsis renal angina index
SCr: Serum creatinine
FB: Fluid balance
