# Supplementary material for: External validation of the modified sepsis renal angina index for prediction of severe acute kidney injury in children with septic shock
Source: Crit Care. 2023 Nov 28;27:463. doi: 10.1186/s13054-023-04746-6 (PMC10683237; doi:10.1186/s13054-023-04746-6)
Supplement: Supplementary file 1 — Additional file 1: Supplemental Methods. Septic shock criteria for original study. [file 13054_2023_4746_MOESM1_ESM.pdf]

Supplemental Methods:

Septic Shock Criteria for Original Study:

(1) At least 2 systemic inflammatory response syndrome (SIRS) criteria (see below) secondary to proven or suspected infection, PLUS
   - Temperature >38 or <36 degrees Celsius
   - Heart rate >90th percentile for age
   - Respiratory rate >90th percentile for age, or hyperventilation to $PaCO_2$ <32 Torr
   - White blood cell count >12,000 or <4,000

(2) Cultures either pending or positive, PLUS

(3) Two distinct measurements of hypotension (mean arterial pressure or systolic blood pressure <3rd percentile for age) after 20 ml/kg of crystalloid or colloid, PLUS

(4) At least one of the following:
   - Requirement of vasoactive support, OR
   - GCS <15 (in the absence of CNS disease), OR
   - Blood lactic acid level >1.6 mmol/L, OR
   - Urine output <1 ml/kg/hr
