# Supplementary material for: External validation of the modified sepsis renal angina index for prediction of severe acute kidney injury in children with septic shock
Source: Crit Care. 2023 Nov 28;27:463. doi: 10.1186/s13054-023-04746-6 (PMC10683237; doi:10.1186/s13054-023-04746-6)

Supplemental Figures:

Figure S1: Patient inclusion CONSORT flow diagram.

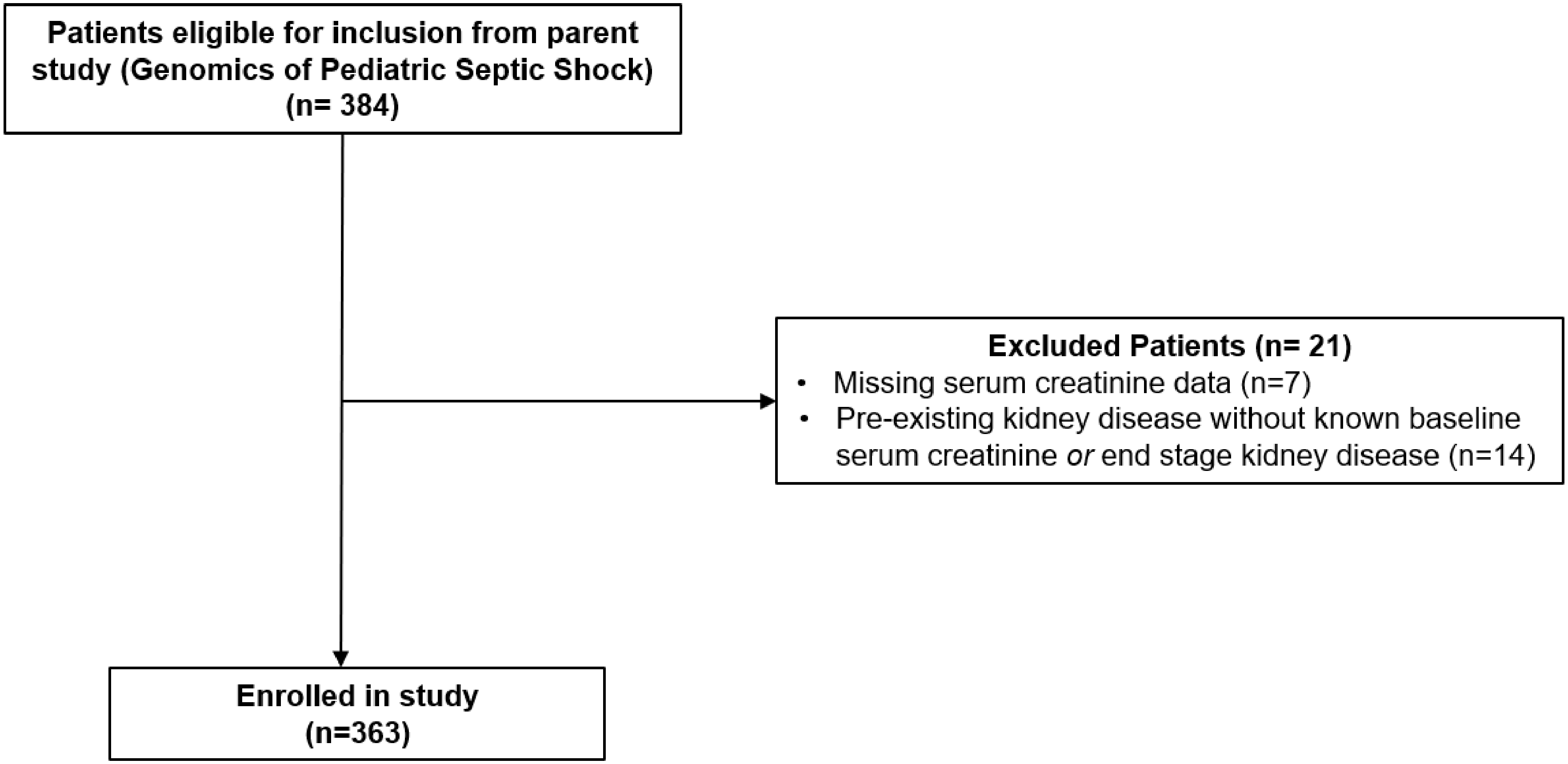

Figure S2: Flow diagram of Day 1 acute kidney injury risk assessment tools and incidence of Day 3 severe acute kidney injury. SCr>Baseline: serum creatinine elevated above baseline; RAI: Renal Angina Index; sRAI: modified sepsis Renal Angina Index.

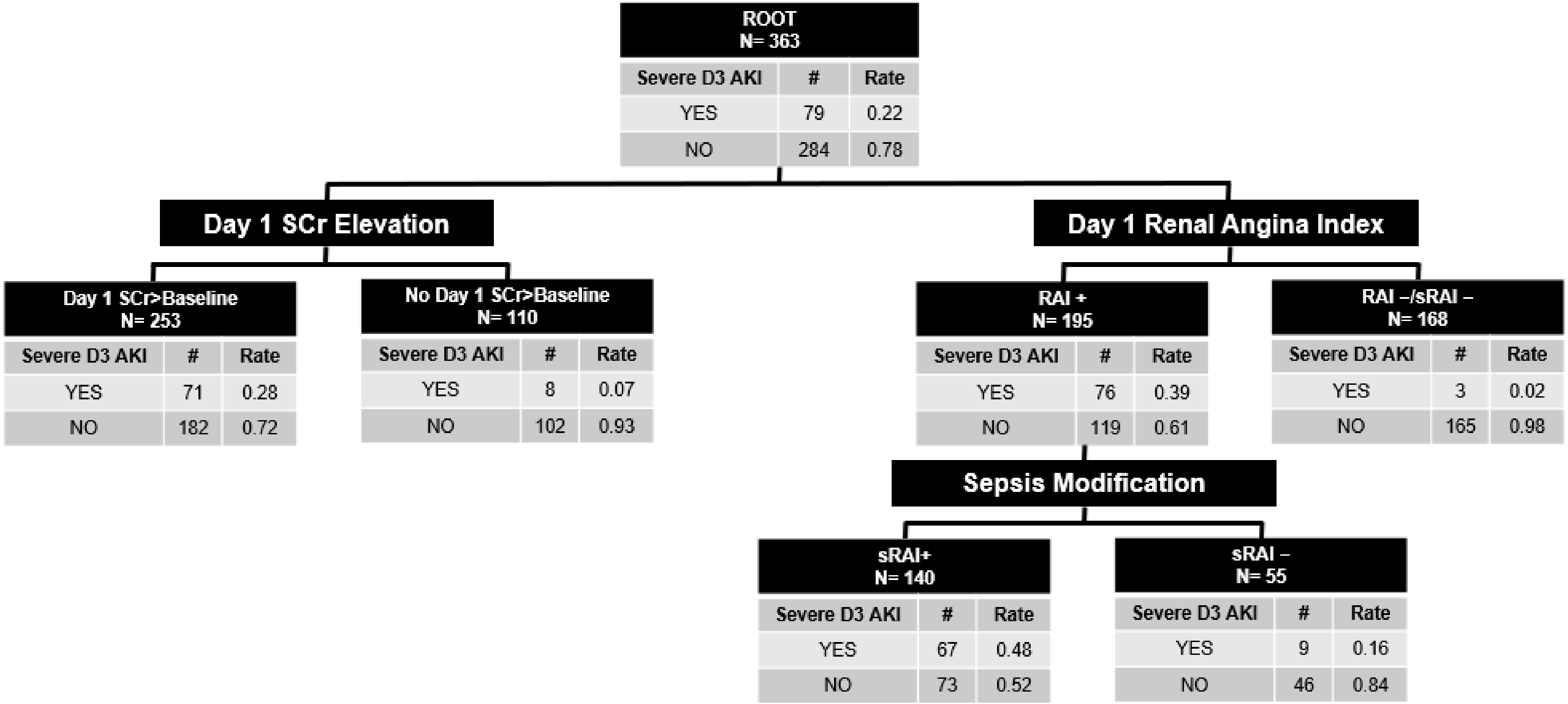

Supplement: Supplementary file 2 — Additional file 2: Supplemental Figures. Figure S1. CONSORT flow diagram for patient inclusion; Figure S2. Flow diagram of Day 1 acute kidney injury risk assessment tools and incidence of Day 3 severe acute kidney injury [file 13054_2023_4746_MOESM2_ESM.pdf]
