# Supplementary material for: External validation of the modified sepsis renal angina index for prediction of severe acute kidney injury in children with septic shock
Source: Crit Care. 2023 Nov 28;27:463. doi: 10.1186/s13054-023-04746-6 (PMC10683237; doi:10.1186/s13054-023-04746-6)
Supplement: Supplementary file 3 — Additional file 3: Supplemental Tables. Table S1. Distribution of outcomes by Renal Angina Index (RAI) Score andmodified sepsis RAI (sRAI) designation; Table S2. Distribution of Outcomes by Renal Angina Index (RAI) and modified sepsis RAI (sRAI) components. [file 13054_2023_4746_MOESM3_ESM.pdf]

Supplemental Tables:

Supplemental Table S1. Distribution of outcomes by Renal Angina Index (RAI) Score and modified sepsis RAI (sRAI) designation.

	Total Patients, n (% cohort)	Incidence D3 Severe AKI, n (% in category)	Incidence D1-7 KRT, n (% in category)
**RAI Score**			
**1**	31 (8.5)	0 (0)	0 (0)
**2**	72 (20)	0 (0)	0 (0)
**3**	2 (0.5)	0 (0)	0 (0)
**4**	33 (9)	2 (6)	2 (6)
**5**	27 (7.5)	1 (4)	1 (4)
**6**	3 (0.5)	0 (0)	0 (0)
**8**	39 (11)	13 (33)	4 (10)
**10**	52 (14)	5 (10)	4 (8)
**12**	4 (1)	1 (25)	0 (0)
**20**	17 (5)	2 (12)	1 (6)
**24**	10 (3)	6 (60)	5 (50)
**40**	73 (20)	49 (67)	32 (44)
**sRAI Designation**			
**Negative**<br>A.RAI <8<br>B.RAI 8 to < 20 *and* platelets ≥ $150x10^3/\mu L$	 168 (46)<br>55 (15)	 3 (2)<br>9 (16)	 3 (2)<br>1 (2)
**Positive**<br>A.RAI Score ≥ 20<br>B.RAI 8 to < 20 *and* platelets < $150x10^3/\mu L$	 100 (28)<br>40 (11)	 57 (57)<br>10 (25)	 38 (38)<br>7 (18)

Data for categorical variables are reported as n (%)

Supplemental Table S2. Distribution of Outcomes by Renal Angina Index (RAI) and modified sepsis RAI (sRAI) components.

RAI/sRAI Components	Total Patients, n (% cohort)	Incidence D3 Severe AKI, n (% in category)	Incidence D1-7 KRT, n (% in category)
**History of Transplantation**			
Yes	34 (9)	18 (53)	13 (38)
No	329 (91)	61 (19)	36 (11)
**D1 Vasoactive Use**			
Yes	268 (74)	70 (26)	45 (17)
No	95 (26)	9 (9)	4 (4)
**D1 Mechanical Ventilation**			
Yes	202 (56)	60 (30)	39 (19)
No	161 (44)	19 (12)	10 (6)
**D1% Fluid Balance**			
<5%	213 (59)	41 (19)	26 (12)
5-10%	103 (28)	21 (20)	14 (14)
10-15%	28 (8)	11 (39)	4 (14)
>15%	14 (4)	6 (43)	5 (36)
**D1 SCr>Baseline**			
Decreased or no change	106 (29)	5 (5)	4 (4)
>1-1.49x,	104 (29)	5 (5)	5 (5)
1.5-1.99x	42 (12)	5 (12)	2 (5)
≥2x	84 (23)	39 (46)	12 (14)
On KRT	26 (7)	25 (96)	26 (100)
**Platelet Count <150 x 10^3/uL**			
Yes	171 (47)	51 (30)	36 (21)
No	192 (53)	28 (15)	13 (7)

Data for categorical variables are reported as n (%)
